# Seizure Occurrence During Hyperbaric Oxygen Therapy: A Case Report and Literature Review

**DOI:** 10.7759/cureus.86391

**Published:** 2025-06-19

**Authors:** Lisle W Blackbourn, Jawad Abou Zeid, Uzair Hamid, Umair Hamid

**Affiliations:** 1 Neurology, University of Illinois College of Medicine Peoria, Peoria, USA; 2 Neurology, OSF HealthCare Illinois Neurological Institute, Peoria, USA

**Keywords:** hyperbaric oxygen therapy, neuronal damage, radiation-induced hemorrhagic cystitis, seizure, tonic-clonic seizure

## Abstract

Pelvic radiotherapy can lead to lasting bladder complications such as radiation-induced hemorrhagic cystitis. Hyperbaric oxygen therapy has emerged as a worthwhile intervention, enhancing tissue oxygenation and promoting neovascularization. However, hyperbaric oxygen therapy carries the risk of seizures, particularly with prolonged exposure to elevated pressures. In this case report, we describe a case of new-onset seizure occurrence in an 88-year-old patient during their first session of hyperbaric oxygen therapy for treatment of radiation-induced hemorrhagic cystitis, highlighting the complexities of balancing therapeutic benefits with potential neurological risks.

## Introduction

Virtually any radiation therapy, including pelvic radiotherapy, can lead to chronic tissue radiation injury [1]. Pelvic radiotherapy, a cornerstone in cancer treatment, may give rise to enduring complications within poorly oxygenated bladder tissues. The underlying mechanism involves progressive endarteritis and chronic fibrosis, triggering tissue hypoxia and anarchic neovascularization [2]. Delayed radiation-induced hemorrhagic cystitis may appear more than 10 years after pelvic radiotherapy [3]. In 1985, Weiss first introduced hyperbaric oxygen (HBO2) therapy as a treatment for post-radiation hemorrhagic cystitis [4]. This approach entails administering oxygen at pressures exceeding atmospheric levels to enhance plasma oxygenation. The expected outcomes involve heightened oxygen levels in hypoxic tissues, fostering neovascularization, mitigation of fibrosis, and enhancing anti-infective defenses [5]. HBO2 therapy is increasingly used for various conditions, including necrotizing soft tissue injuries, refractory osteomyelitis, severe anemia, and carbon monoxide poisoning.

HBO2 therapy is typically administered at pressures ranging from 2 to 2.5 atmospheres absolute (ATA) to achieve therapeutic effects. Furthermore, the efficacy of HBO2 therapy for hemorrhagic cystitis at a pressure below 2 ATA is not well established [6]. In fact, lower pressures may not provide sufficient oxygenation to effectively treat hemorrhagic cystitis. Systematic review has shown that common side effects of HBO2 therapy are usually self-limiting and that serious side effects, such as seizure, are rare [6].

Here we present a case of an 88-year-old male with hemorrhagic cystitis as a suspected complication of his radiation therapy for prostate cancer who presented with first-time seizure activity after HBO2 therapy. We use this case to help evaluate the balance between the potential benefits and risks of HBO2 therapy while discussing options for those who have seizure activity due to HBO2 therapy.

## Case presentation

The patient is an 88-year-old male with a past medical history of heart failure, prostate cancer treated with radiation therapy and chemotherapy, hypertension, hyperlipidemia, coronary artery disease, and hypothyroidism who presented to the emergency department after an episode of seizure-like activity. The patient had been recently diagnosed with hemorrhagic cystitis as a suspected complication of his radiation therapy. The patient had been set up for monoplace sessions of HBO2 therapy to treat his hemorrhagic cystitis by his urologist.

During the first treatment set at a pressure of 2.4 ATA, the patient had witnessed generalized tonic-clonic seizure activity during the second air break while in the chamber by the staff there that lasted approximately one minute. The second air break was 60 minutes into his treatment session. The patient was reported to have some confusion following the episode that lasted somewhere between 30 minutes and 60 minutes. The patient did not have any tongue biting or urinary incontinence. The patient denied any prior seizure history. The patient was at his baseline when evaluated by neurology hours after the event and had no lateralizing or focal neurologic deficits.

**Figure 1:**
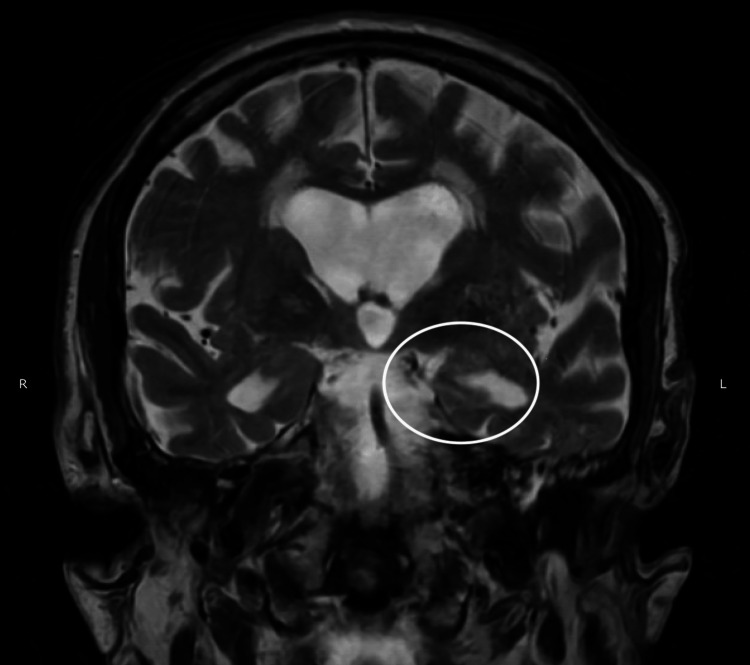
MRI brain with and without contrast coronal view The MRI (Magnetic Resonance Imaging) image is showing asymmetric prolonged T2 values at the left greater than the right hippocampal regions.

Initial laboratory work was unremarkable except for an elevated lactate at 4.6 mmol/L (normal range 0.7-2.0 mmol/L). A CT head showed no acute intracranial abnormalities. An overnight EEG showed occasional focal sharp waves in the left temporal region, consistent with potentially epileptogenic focal cortical hyperexcitability in that region. An MRI brain with and without contrast showed an element of asymmetric prolonged T2 values at the left greater than right hippocampal regions (Figure 1).

The patient was initiated on levetiracetam 500 mg twice a day with recommendations to discuss lowering the pressure for his HBO2 therapy with his urologist. The neurologist discussed that he could continue HBO2 therapy sessions.

## Discussion

While HBO2 therapy is generally acknowledged as safe, seizures, usually described as brief, oxygen-related generalized tonic-clonic convulsions typically occurring towards the end of a treatment session or prior to a schedule air break, exhibit conflicting incidence rates, ranging from one per 10,000 to one per 600 seizures per hyperbaric sessions [7-9]. However, the mechanism behind this complication is poorly understood [7].

The concept that oxygen might trigger hyperactivity in the central nervous system was first introduced in 1878 [10]. In fact, inhalation of hyperbaric oxygen can lead to grand mal seizures attributed to oxygen toxicity, regardless of whether symptoms precede the seizure event. The intensity of CNS hyperactivity and the likelihood of seizure occurrence are dependent upon the partial pressure of oxygen and the duration of exposure [8]. Although the precise mechanism underlying CNS oxygen toxicity remains uncertain, one proposed explanation involves the production of reactive oxygen species during HBO2, resulting in the peroxidation of membrane lipids and the modulation or inhibition of enzymes, thereby altering brain metabolism and electrical activity [11].

While there is no conclusive evidence linking seizures to oxygen-induced metabolic alterations, there is an observed increase in glucose utilization immediately before the manifestation of electrophysiological signs of CNS oxygen toxicity [8]. Additionally, another proposed mechanism involves elevated levels of nitric oxide in the brain, potentially leading to vasodilation in cerebral vessels, and subsequently enhancing oxygen delivery to the brain [11].

Recent reports have shown a statistically significant increased risk of oxygen toxicity at higher treatment pressures [7]. For instance, Banham N demonstrated a significantly high risk of oxygen-related seizures at pressures above 203 kPa (2 ATA) (odds ratio (OR) 8.5, 95% confidence interval (CI) 2.0 to 36.1; P<0.001) [7]. This risk was evidently higher as treatment pressure increased from 2 to 2.4/2.5 ATA among previously healthy individuals (OR 5.1, 95% CI 1.1 to 22.8; P<0.028). While several studies have collectively underscored the valuable therapeutic efficacy of HBO2 therapy, typically administered at pressures ranging from 2 to 2.5 ATA in the reduction of hematuria severity and improvement of bladder function; nevertheless, a randomized double-blinded trial compared the efficacy of HBO2 therapy at a high pressure of 2.4 ATA versus a lower pressure of 1.3 ATA in patients with hemorrhagic cystitis and found that higher pressures resulted in a sustained decrease of cystitis symptoms compared to lower pressures that did not result in a significant improvement of baseline parameters [12-14]. One study had no seizures at a pressure of 2 ATA in their research population [9]. In our patient, treatment was set at a pressure of 2.4 ATA. Due to the data above, it would have been reasonable to try lowering the pressure along with adding a seizure medication to continue to treat his hemorrhagic cystitis if the patient had chosen to continue with treatments.

Furthermore, over the last three decades, emerging research has suggested that even brief seizures could potentially inflict damage to the brain. Sporadic brief seizures induced by triggering stimulation of limbic structures led to neuronal loss in the hippocampal formation [15]. Similarly, neuronal apoptosis in the hippocampus has been seen following a single induced seizure [16]. Another study demonstrated that repeated brief seizures in rats resulted in subfield-specific hippocampal neuronal loss and deficits in spatial memory function, resembling hippocampal sclerosis seen in temporal lobe epilepsy cases [17]. Tissue damage was triggered via apoptotic or necrotic pathways, depending on the time course of the injury and cellular type in rat models [18]. As such, research revealed heightened levels of apoptotic markers in the hippocampus a week post-hyperbaric induced-seizure, which indicates apoptosis occurrence, possibly mediated by mitochondrial involvement [19]. The underlying mechanism by which HBO2-induced seizures trigger apoptosis remains uncertain, although reactive oxygen species, known to induce apoptosis and are elevated during HBO2-induced seizures, present a plausible avenue for further exploration [19]. There have also been reports of stroke caused by HBO2-induced seizures [20].

Such findings would aim to prompt a discussion regarding the conditions under which the perceived benefits of employing higher treatment pressures outweigh the documented increase in the risk of oxygen toxicity seizures. In our patient, the EEG showed discharges corresponding to the same region of asymmetry on the MRI scan of the brain. Although no events were captured on EEG, given the EEG findings in correlation with the MRI and an elevated lactate level, it was highly suspicious that this was a true seizure event versus a non-epileptic event. It also possibly suggested that the patient could be at risk of a second seizure event, regardless of HBO2 therapy continuation, given the location of MRI asymmetry coupled with EEG findings. It is suspected that HBO2 lowered the patient’s abnormal seizure threshold, rather than this being a true oxygen-toxic seizure. MRI also ruled out other causes of seizure in our patient’s age group, such as structural lesions like tumors or stroke, or an infectious cause like encephalitis. Further HBO2 therapy in our patient was declined after the patient had discussions with his urologist.

## Conclusions

A significant increase in reported seizures with escalating treatment pressure underscores the importance of carefully assessing the risk-benefit profile of HBO2 therapy across various protocols and the development of standardized treatment guidelines versus considering tailoring the treatment to the individual patient if they have factors that may increase the risk of seizure. Informed decision-making regarding HBO2 therapy should prioritize discernible benefits while acknowledging the inherent risks associated with heightened treatment pressures.
